# Association between the Urinary Bladder Volume and the Incidence of “De Novo” Overactive Bladder in Patients with Stress Urinary Incontinence Subjected to Sling Surgeries or Burch Procedure

**DOI:** 10.1155/2019/9515242

**Published:** 2019-02-12

**Authors:** Sylwester Ciećwież, Kornel Chełstowski, Agnieszka Brodowska, Magdalena Ptak, Dariusz Kotlęga, Andrzej Starczewski

**Affiliations:** ^1^Department of Gynecology, Endocrinology and Gynecologic Oncology, Pomeranian Medical University in Szczecin, Poland; ^2^Chair of Medical Analytics, Department of Laboratory Diagnostics, Pomeranian Medical University in Szczecin, Poland; ^3^Department of Medical Rehabilitation and Clinical Physiotherapy, Pomeranian Medical University in Szczecin, Poland; ^4^Department of Neurology, Pomeranian Medical University in Szczecin, Poland

## Abstract

*Aim. *The aim of the study was to compare the incidence of “de novo” overactive bladder (OAB) after sling surgeries and Burch procedure and to analyze the effect of the preoperative bladder volume on the incidence of this condition.* Methods. *This prospective trial included 290 female patients with stress urinary incontinence (SUI) who were subjected to sling surgeries (TOT or TVT, n=170) or Burch procedure (n=120). Urodynamic testing was performed prior to the surgery and 6 months thereafter. The presence of OAB was diagnosed on the basis of subjective symptoms and urodynamic parameters.* Results. *The incidence of OAB 3 at 6 months postsurgery was the highest in patients who were subjected to the Burch procedure (14.2% and 17.5%, respectively). The incidence of OAB at 6 months turned out to be significantly higher in patients subjected to the Burch procedure with preoperative bladder volumes greater than 353 ml. We observed the significant postoperative decrease in the bladder volume of women who developed this complication following the Burch procedure.* Conclusions. *Among surgeries for stress urinary incontinence, Burch procedure is associated with the greatest risk of overactive bladder development. Probably, one reason for the higher incidence of overactive bladder after Burch procedure is the intraoperative reduction of the urinary bladder volume.

## 1. Introduction

Stress urinary incontinence (SUI) is defined as an unintentional, uncontrolled loss of urine through the urethra. This happens when vesical pressure exceeds the urethral sphincter pressure during coughing, sneezing, or physical exercise. The presence of SUI does not alter the frequency of micturition during the day and night and, contrary to the overactive bladder, is not associated with an urgent need to urinate [1].

According to the ICS definition published in 2002, overactive bladder (OAB) is a multisymptomatic disorder manifested with sudden urgency with or without urinary incontinence. It is usually associated with an increased frequency of micturition during the day and nocturia. Polyuria is currently defined as a subjective impression of excessive urination during the day, and nocturia as more than one micturition during the night [2–4]. Importantly, nocturia can be diagnosed only if one had to wake at night for voiding and fell asleep afterwards [1, 5–19]. It needs to be stressed that OAB can be diagnosed only after exclusion of other factors explaining the abovementioned symptoms, such as urinary tract infection, interstitial cystitis, tumors, urolithiasis, and neurological disorders [20]. Due to such definition of OAB, diagnosis of this condition does not necessitate urodynamic testing. Nevertheless, the evidence of phasic or terminal detrusor overactivity (i.e., an increase in the detrusor pressure above 15 cm H_2_O) constitutes an objective diagnostic criterion of OAB. It needs to be stressed that only approximately 40-60% of patients with the OAB-characteristic symptoms present with the detrusor overactivity on urodynamic testing [21]. The latter is usually accompanied by other, less characteristic symptoms, such as a sudden increase in the vesical pressure after administration of even small amount of infusion fluid, increase in myogenic activity, and spastic or terminal contractions. The presence of overactive bladder is not necessarily associated with urinary incontinence. Therefore, an urgent need to urinate with resultant urinary incontinence is classified as the “wet” OAB, whereas the sudden urgency without urinary incontinence is referred to as the “dry” OAB.

In this study, we focused on the incidence of “de novo” overactive bladder as a complication of sling surgeries (TOT and TVT) and Burch procedure. These procedures constitute the most popular methods of surgical treatment of stress urinary incontinence, and overactive bladder represents one of their most prevalent complications, markedly diminishing quality of life during postoperative period. Sling surgeries and Burch procedure are equally efficient in the treatment of SUI. The incidence of OAB after these procedures ranges between 3% and 66% [22], but according to some authors may reach even up to 75% [23]. If “de novo” sudden urgency is observed immediately after the surgery, one should exclude its other potential causes, such as incorrect placement of tension-free tape, e.g., inside the bladder or too close to its neck, postmicturition urinary retention, and urinary tract infection [24]. Similarly, in the case of late postoperative manifestation of OAB-like symptoms, a number of other potential causes should be considered. Finally, it needs to be emphasized that in many patients who present with overactive bladder after surgical treatment of stress urinary incontinence, the exact etiology of OAB remains unknown.

Due to their established high effectiveness, sling surgeries and Burch procedure are most common surgical treatments of SUI [25–27]. We hypothesized that selection of the most adequate surgical procedure for given patient might not only maximize the effectiveness of SUI treatment, but also minimize the risk of postoperative OAB. Therefore, the aim of the study was to compare the incidence of “de novo” OAB after sling surgeries and Burch procedure and to analyze the effect of the bladder volume on the incidence of this condition.

## 2. Materials and Methods

This prospective trial included 290 female patients who were operated on due to pure SUI in the Department of Fertility and Gynecology, Pomeranian Medical University in Szczecin. The presence of SUI was confirmed on the basis of a survey with the Gaudenz questionnaire [28], physical examination, and urodynamic testing. The study group was predominated by patients qualified to sling surgeries: TOT or TVT (n=170, 58.62%). The exact type of the sling procedure was chosen on a random basis. The remaining 120 patients (41.38%) were qualified to Burch procedure. The indication to the latter procedure was necessity of simultaneous laparotomic resection of benign endometrial or adnexal lesions.

The inclusion criterion of the study was presence of pure grade II or III SUI confirmed on urodynamic testing. The exclusion criteria were (1) preoperative evidence of OAB and mixed urinary incontinence, (2) grade I SUI, (3) stage 3 or 4 genital prolapse, (4) pre- and/or postoperative retention of more than 50 ml of urine and bladder outlet obstruction documented on urodynamic testing, (5) presence of early or late, direct postoperative complications, (6) incorrect placement of TOT or TVT tape confirmed on postoperative urogynecological ultrasonography, (7) history of surgical treatment for SUI, (8) urinary fistulae, (9) congenital and/or acquired defects of the urethra and bladder, (10) urinary tract infections, (11) hormone replacement therapy prior to and after the procedure, (12) cardiovascular conditions (arterial hypertension, ischemic heart disease, arrhythmia), (13) type 1 or 2 diabetes mellitus, and (14) deformation of the lumbosacral spine (spina bifida, posttraumatic and/or degenerative lesions).

Shortly before the surgery the patients were surveyed with the Gaudenz questionnaire [28] and subjected to gynecological examination and urodynamic testing with determination of the bladder volume. Urodynamic testing was performed after confirming normal result of urinalysis. All urodynamic tests were performed by the same investigator. The MMS Libra System (Medical Measurement Systems B.V.) was used to determine vesical (P_ves_), abdominal (P_abd_), and detrusor (P_det_) pressure. Moreover, the first sensation of bladder filling (FS), first desire to void (FD), and strong desire to void (SD) were recorded during the filling phase. Also cough stress test and Valsalva maneuver were performed after administering the first 50 ml of fluid and repeated after every additional 100 ml. The micturition phase started after maximum filling of the bladder. The patient voided to a container placed on a scale, and uroflow curve and residual urine volume were determined. The presence of OAB was diagnosed on the basis of subjective symptoms reported by a patient and such urodynamic parameters as reactivity of the bladder, FS, ND, SD, and detrusor pressure. The survey with the Gaudenz questionnaire was repeated 3 and 6 months postsurgery, and urodynamic testing with determination of the bladder volume was performed at 6 months.

The protocol of the study was approved by the Local Bioethics Committee at the Pomeranian Medical University (decision no. BN-001/16/06), and written informed consent was obtained from all the participants.

Statistical analysis was conducted with Statistica 10 package (StatSoft, United States). Statistical characteristics of continuous variables were presented as arithmetic means, standard deviations, medians, minimum and maximum values, and the characteristics of qualitative variables as numbers and percentages. The normality of continuous variable distribution was verified with Shapiro-Wilk test. The effects of surgery type were compared with a two-way repeated measure ANOVA surgery (3: TOT, TVT, Burch procedure) x time (2: prior to surgery and 6 months thereafter). The Tukey's post hoc test was used for multiple comparisons. Due to small sample size, relationships between pairs of qualitative variables were tested with the Pearson's chi-square test with Yates' correction or Fisher exact test. Moreover, some continuous variables were transformed into discrete variables on the basis of their median values. The threshold of statistical significance of all the tests was set at p≤0.05.

## 3. Results

**Figure 1:**
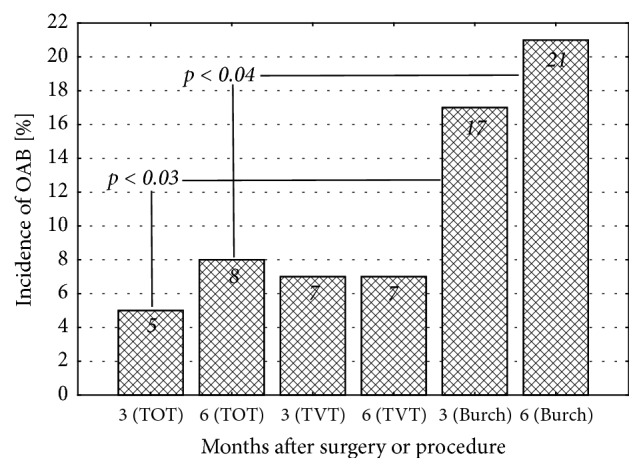
Incidence of overactive bladder 3 and 6 months after sling surgeries (TOT and TVT) and Burch procedure.

The incidence of overactive bladder 3 and 6 months postsurgery was the highest in patients who were subjected to Burch procedure (14.2% and 17.5%, respectively) and the lowest in women operated on according to TOT protocol (4.6% and 7.4%, respectively). The incidence of OAB in these two groups differed significantly at both three (p=0.03) and six months (p=0.04) postsurgery. The incidence of OAB in patients operated on according to TVT protocol did not differ significantly when compared to the remaining groups (Figure 1).

**Figure 2:**
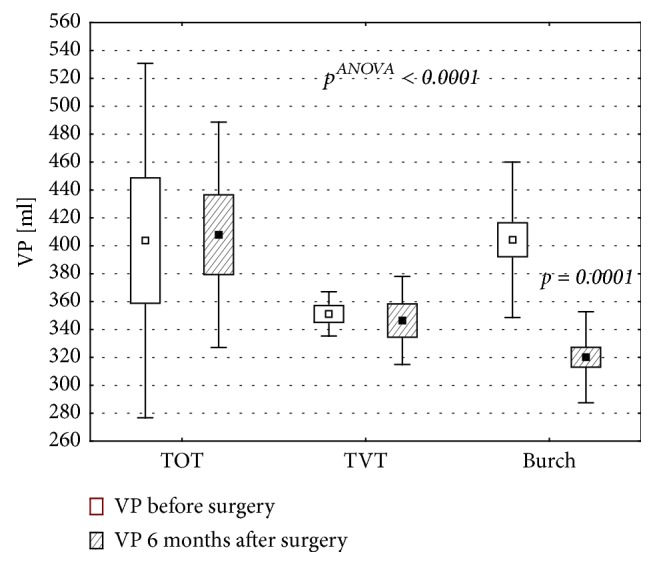
Mean urinary bladder volume of patients who developed OAB, determined prior to a sling surgery or Burch procedure and 6 months thereafter. Vp: urinary bladder volume; p-value of the Tukey's test.

In all patients, the urinary bladder volume prior to the surgery and 6 months thereafter ranged between 102 ml and 636 ml. While the patients who developed OAB after TOT or TVT surgeries did not show significant postoperative changes in the bladder volume, we observed the significant decrease in the bladder volume of women who developed this complication following Burch procedure (p=0.0001, Figure 2).

**Figure 3:**
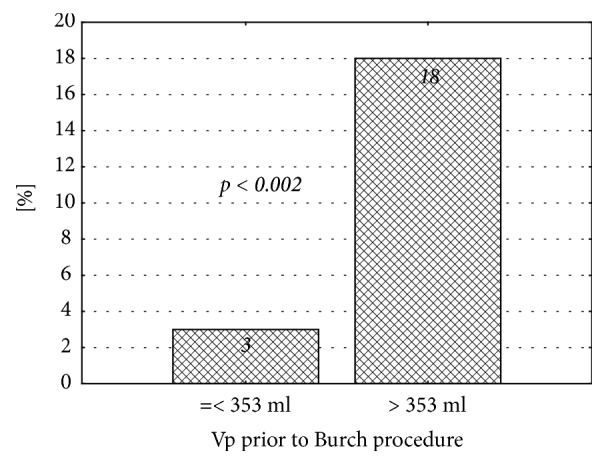
Incidence of overactive bladder 6 months after Burch procedure, stratified according to the preoperative urinary bladder volume.

Six months postsurgery, the incidence of OAB in patients who were subjected to Burch procedure with preoperative bladder volumes greater than 353 ml turned out to be significantly higher than in the remaining women operated on with the same surgical technique (Figure 3). We did not document similar relationships in the TOT- and TVT-operated patients, but the size of these two subgroups might be too small to draw any firm conclusions.

**Figure 4:**
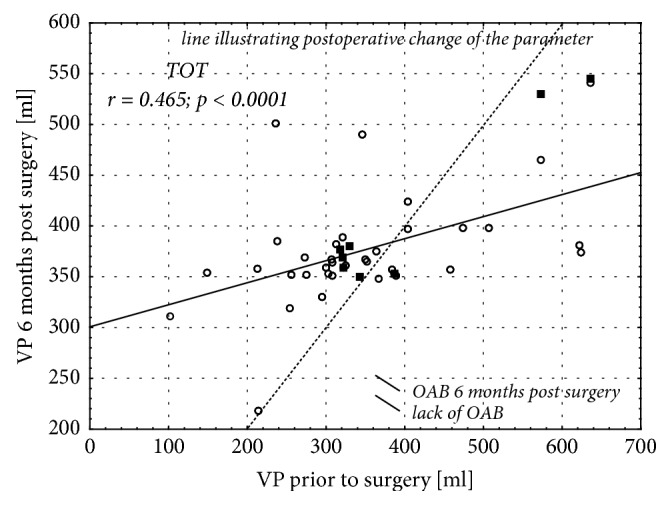
Correlation between urinary bladder volumes determined prior to surgery according to TOT protocol and 6 months thereafter.

**Figure 5:**
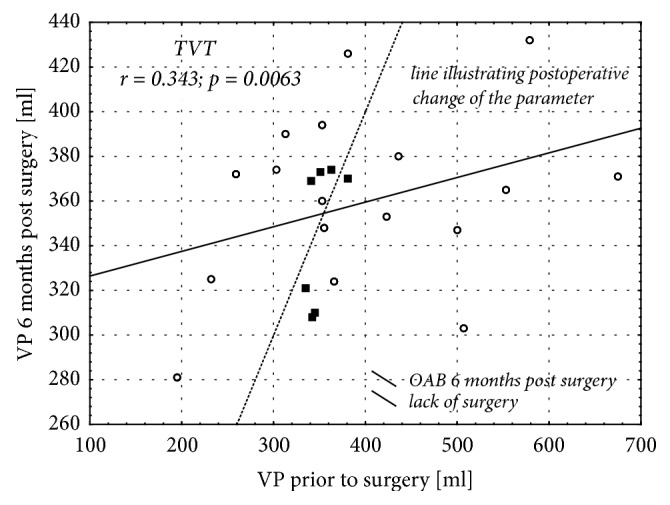
Correlation between urinary bladder volumes determined prior to surgery according to TVT protocol and 6 months thereafter.

**Figure 6:**
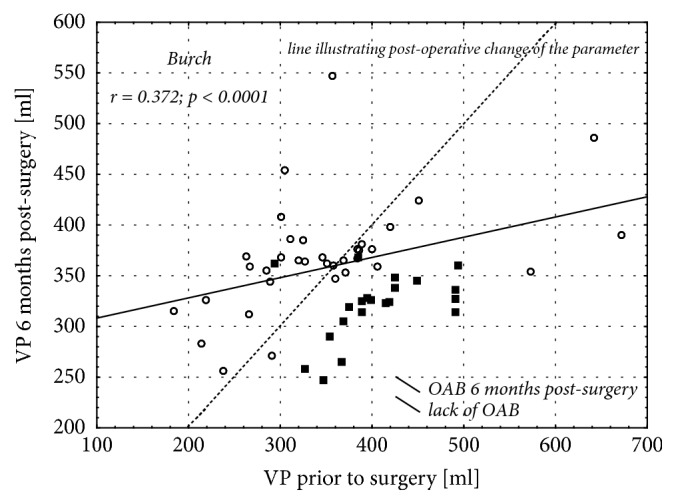
Correlation between urinary bladder volumes determined prior to Burch procedure and 6 months thereafter.

Analysis of correlation between the pre- and postoperative bladder volumes showed only slight postoperative changes of this parameter or complete lack thereof in most of the women who developed OAB following a sling surgery (Figures 4 and 5). In contrast, a postoperative decrease in the urinary bladder volume was observed in all but two patients who developed OAB as a consequence of Burch procedure (Figure 6).

## 4. Discussion

TOT and TVT surgeries and Burch procedure constitute currently the most popular methods for surgical treatment of SUI. Regardless of the surgical method, the outcomes of the treatments are good. The effectiveness varies according to the follow-up time, from approximately 90% after one year to ca. 70% at five years after the surgery [25–27]. Unfortunately, some patients operated on for SUI may develop OAB which is detrimental for their quality of life in many dimension. It is unclear whether presence of SUI impairs quality of life to a larger extent than OAB with severe nocturia. Our study revealed that the incidence of OAB, both 3 and 6 months postsurgery, was the highest after Burch procedure (14.2% and 17.5%, respectively). The incidence of OAB documented 3 and 6 months after TVT surgery was similar, amounting to ca. 11%. The complication turned out to be least frequent 3 and 6 months after TOT procedure, amounting to 4.6% and 7.4%, respectively. According to other authors, OAB may occur in 3-66% [22] or even 75% of patients after surgical treatment of SUI [23].

Due to specific structure of its wall, urinary bladder is highly flexible and can substantially change its volume. According to various authors, the volume of the urinary bladder ranges between 350 ml and 650 ml [1]. Theoretically, the factors that impair the bladder's flexibility may also interfere with the detrusor function [29]. Only few authors analyzed the influence of the surgical treatment of SUI on the bladder volume and relationship between postoperative changes in this latter parameter and incidence of OAB. A subanalysis of postoperative changes in the bladder volume of our patients who developed OAB 6 months postsurgery showed a significant decrease in this parameter of women who were subjected to Burch procedure. We did not observe this relationship in the remaining subgroups of patients. Moreover, we showed that larger volume of the bladder prior to Burch procedure was associated with significantly higher incidence of OAB 6 months after the surgery. Similar association was not documented in the case of women who were operated on according to TOT and TVT protocols. Probably, the patients whose bladder volume was significantly reduced as a result of Burch procedure were more likely to develop “de novo” OAB after the surgery. Also the fact that this procedure is associated with a reduction of the bladder volume at the level of its neck and resultant change of the urethrovesical angle, is worth noticing. This hypothesis seems to be supported by our finding on lower incidence of this complication after TOT and TVT surgeries. To the best of our knowledge, none of the previous studies analyzed a relationship between the bladder volume and incidence of “de novo” OAB. Wang et al. [30] compared the outcomes of TVT and Burch surgeries in patients with stress urinary incontinence and analyzed the influence of these procedures on the bladder volume and residual urinary volume. However, they did not analyze the association between the bladder volume and incidence of OAB. Instead, they only showed that the bladder volume of patients subjected to Burch procedure decreased significantly, contrary to persons who were operated on according to TVT protocol [30]. The treatment outcomes in patients with stress urinary incontinence were also studied by Han et al. [31]. Among the parameters analyzed by these authors were the bladder volumes determined one month, one year, and five years after the procedure. The authors did not document significant changes in the bladder volume of patients subjected to TVT surgery [31]. Our findings seem to be consistent with this observation. Overall, these data point to a necessity of preoperative assessment of the bladder volume in patients with SUI and appropriate selection of the surgery type in order to prevent excessive postoperative reduction of this parameter.

## 5. Conclusions

Among surgeries for SUI, Burch procedure is associated with the greatest risk of overactive bladder development. Probably, one reason for the higher incidence of overactive bladder after Burch procedure is the intraoperative reduction of the urinary bladder volume.

## Data Availability

Dataset connected to this study is available from the corresponding author upon a reasonable request.
